# Progressive Visual Loss Without Retinal Detachment in Stickler Syndrome: An Uncommon and Novel Presentation

**DOI:** 10.4274/tjo.galenos.2020.33858

**Published:** 2020-12-29

**Authors:** Ana Navarrete, Adva Kimchi, Jaime Levy, Vardiella Meiner, Radgonde Amer, Claudia Yahalom

**Affiliations:** 1 Hadassah-Hebrew University Medical Center, Department of Ophthalmology, Jerusalem, Israel; 2 Hadassah-Hebrew University Medical Center, Department of Genetics and Metabolic Diseases, Jerusalem, Israel

**Keywords:** Stickler syndrome, myopia, retinal atrophy

## Abstract

Stickler syndrome is known to cause visual handicap due to the high incidence of retinal detachment. We aim to present an unusual case of a child with Stickler syndrome who had progressive visual loss secondary to atrophy of the outer retinal layers not associated with retinal detachment. This is a descriptive case report of a 9-year-old child with ocular history of high myopia who presented to our institution with suboptimal visual acuity in both eyes. After 2 years of follow up, he developed unilateral progressive visual loss with marked atrophy of the outer retinal layers and peripheral vascular leakage. Such a presentation has not been previously described in the literature to the best of our knowledge.

## Introduction

Stickler syndrome is a hereditary connective tissue disorder associated with ocular, orofacial, musculoskeletal, and auditory manifestations. It is the most common inherited vitreoretinopathy, estimated to affect 1 in 7,500 to 9,000 newborns.^1^

Mutations in several genes cause the different types of Stickler Syndrome. The autosomal dominant types are Stickler type I, which is due to a mutation in *COL2A1* and accounts for 80-90% of cases; Type II, which is caused by mutation in *COL11A1* and accounts for 10-20% of cases; and Type III, which occurs due to a mutation in *COL11A2* and is characterized by non-ocular manifestations. The autosomal recessive types include Stickler type IV and V with mutations in the *COL9A1* and *COL9A2* genes, respectively.

The most common ocular manifestations are high myopia and vitreous syneresis (100% of patients). Stickler type I is characterized by membranous vitreous and type II by beaded, fibrillar vitreous.^2^ Vitreous veils attached to the retina, radial perivascular atrophy, and retinal lattice degeneration are also common. Retinal detachments secondary to anterior giant retinal tears or posterior breaks are common, as well as pre-senile cataract.^3^

We present the case of a boy with high myopia and progressive visual loss not related to retinal detachment. After an exhaustive investigation including whole exome sequencing (WES), Stickler syndrome type I was diagnosed, with unusual ophthalmological findings not previously described in the literature.

## Case Report

A 9-year-old patient was referred to our clinic due to suboptimal visual acuity. He had ocular history of high myopia, as did his father and grandfather.

At presentation, logMAR best corrected visual acuity (BCVA) was 0.48 in the right eye (RE) and 0.18 in the left eye (LE). The refraction was RE -7.50 -1.00 x180 and LE -7.50 -0.75 x180. On eye examination, the anterior segments were normal and the vitreous was quiet, with a vitreous strand overlying the superotemporal retina in the LE. The retina was flat with fine macular and perivascular pigmentary changes (RE more than LE).

**Figure 1:**
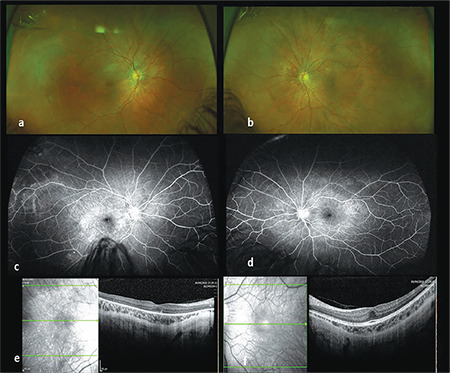
Auxiliary tests at presentation. a, b) Color fundus images (Optos 200 Tx, Optos PLC, Dunfermline, United Kingdom) of the right and the left eye respectively show prominence of the choroidal vessels around the optic discs and in the macular areas. c, d) Fluorescein angiography (Optos 200 Tx, Optos PLC, Dunfermline, United Kingdom) showed an area of hyperfluorescence around the optic discs and in the macular areas in both eyes and in the temporal peripheral retina of the right eye. e, f): Spectral domain optical coherence tomography (SD-OCT, Heidelberg Engineering, Heidelberg, Germany) showed an irregular area of the retinal pigmented epithelium in the right eye with attenuated outer retinal bands and decreased foveal pit bilaterally. Central macular thickness: 214 μm right eye; 295 μm left eye

Figure 1 summarizes additional test findings including spectral domain optical coherence tomography (SD-OCT) and fluorescein angiography (FA). Bilateral foveal hypoplasia was noted with attenuation of outer retinal bands in the RE and hyperfluorescence in the macular areas bilaterally. Electroretinogram (ERG) showed nonspecific decreased mixed cone-rod response.

**Figure 2:**
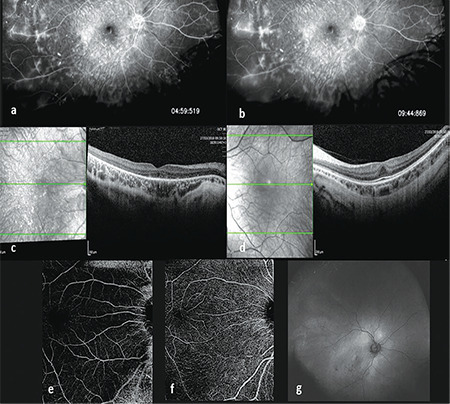
Auxiliary tests 3 years later. a, b) Fluorescein angiography of the right eye showing leakage of the temporal peripheral vessels and some mottling in the nasal peripheral retina. c, d) Spectral domain optical coherence tomography (SD-OCT) of the right and left eye respectively shows total disruption of outer retinal bands and atrophy of outer nuclear layer in the right eye. SD-OCT of the left eye shows foveal hypoplasia; no atrophy of the outer retinal bands was observed. Central macular thickness: 198 μm right eye; 292 μm left eye. e, f) OCT-angiography (AngioPlex Elite 9000, Carl Zeiss Meditec, Inc., Dublin, USA): superficial capillary plexus and deep capillary plexus were within normal limits. Choroidal thickness at the fovea in the right eye was 227 μm (1000 μm temporal: 182 μm, 1000 μm nasal: 221 μm, and maximal choroidal thickness was at 3000 μm temporal to the fovea, measuring 276 μm). In the left eye, choroidal thickness at the fovea was 175 μm (1000 μm temporal: 190 μm, 1000 μm nasal: 174 μm, maximal choroidal thickness was also at 1000 μm temporal to the fovea, 190 μm. g) Fundus autofluorescence of the right eye shows increased autofluorescence around the optic disc and tiny areas of hypoautofluorescence at the posterior pole

Two years later, BCVA had decreased considerably to 1.0 logMAR in his RE and remained stable in his LE. During this examination there was evidence of bilateral marked vitreous syneresis with membranous formations. There were no vitreous cells and retinal findings remained unchanged. Repeated FA showed leakage from the peripheral vessels in the RE and focal areas of capillary nonperfusion. Fundus autofluorescence showed areas of hypoautofluorescence in the posterior pole. SD-OCT demonstrated total loss of the ellipsoid zone and marked atrophy of the outer retinal layers in the RE. The LE remained stable. Swept source OCT-angiography showed no abnormal vascularization (Figure 2). Repeated ERG examination evidenced worsening of cone-rod function.

The child was referred for genetic testing. WES revealed a frame-shift pathogenic variant (c.2807_2810dupGCCC; p.Gly939ProfsTer6) in exon 42 of the *COL2A1* gene, which suggested the diagnosis of Stickler syndrome type I. His parents were tested by Sanger sequencing for the genetic variant and were not found to carry the variant, indicating that it occurred as a de novo mutation in the child. WES was repeated by a laboratory specialized in inherited retinal diseases in order to rule out additional mutations that can explain a retinal dystrophy in this child, but no other mutations were identified.

The original anamnesis reported that the child was born with bifid uvula, and also described some mild orthopedic problems. Physical examination by a clinical geneticist following genetic tests results showed very subtle signs of malar hypoplasia with retromicrognatia and crowded teeth, bifid uvula and high arched palate, and camptodactyly of the fifth finger. These findings supported the diagnosis of Stickler syndrome.

## Discussion

Stickler Syndrome is a rare hereditary connective tissue disease. Clinical manifestations and targeted genetic testing are generally sufficient to reach a diagnosis. Most cases are inherited via autosomal dominant inheritance, while a minority of cases result from de novo mutations, as in our case.^4^ Non-ocular findings can include incomplete palate, which ranges from open cleft, submucous cleft, to bifid uvula like in our case. Hearing loss, joint hypermobility, and other skeletal manifestations are also seen.^3,5^

When systemic signs are not evident, ophthalmologists play a major role in the diagnosis. This occurs especially in cases of mutations in exon 2 of the *COL2A1* gene that can produce a phenotype with predominantly ocular manifestations.^1,6^ The majority of patients presenting to an ophthalmologist will have either type 1 or type 2 Stickler syndrome and are frequently high myopes.^7^

Our patient presented with progressive disruption of the outer retinal layers leading to visual loss in one eye. Peripheral vascular leakage (retinal capillaritis) and possible thick choroid were also detected. These changes have not been previously described in Stickler syndrome and may be the result of mild vascular changes. Retinal capillaritis has been previously described in the setting of CRB1-associated retinal dystrophy; the authors suggested that capillaritis may be due to the active phase of the disease in young patients, although the influence of modifier genes could not be excluded.^8^

Our patient also presented with radial perivascular pigmentary degeneration which is known to be a characteristic manifestation of Stickler that develops in childhood and progresses with time.^2,6^ Abnormal ERG with progressive abnormalities of cone-rod function was seen in our patient and has already been described in Stickler Syndrome.^1^

Our patient also presented with bilateral foveal hypoplasia, with good vision in the LE. Recently, foveal hypoplasia has been associated with Stickler syndrome.^9,10^ In 2018, Matsushita et al. studied the degree of foveal hypoplasia in patients diagnosed with Stickler syndrome type I and found that 82% of the subjects had mild foveal hypoplasia with persistence of the inner retinal layers in the fovea in OCT images.^9^

Foveal hypoplasia had not been commonly reported in patients with Stickler syndrome probably because these patients have fairly good visual acuity.^11^ Recent advancements and accessibility of high-resolution OCT imaging have shown that a lack of foveal pit does not always indicate poor visual acuity.

Visual loss and blindness in children with Stickler syndrome has classically been related to the presence of retinal detachment.^2,11^ In our case, there was progressive visual loss secondary to total loss of the ellipsoid zone and outer retinal layer atrophy without retinal detachment, not previously described in Stickler cases. Other possible additional diagnoses such as posterior uveitis, infection, and retinal dystrophy were ruled out, raising the suspicion that this retinal atrophy was not a coincidental finding but a potential Stickler-related ocular manifestation not previously reported.

WES is a useful tool that assists ophthalmologists in reaching the correct clinical diagnosis and ruling out additional genetic pathology in complex cases.
